# Long Versus Short Twin-Screw Integrated Cephalomedullary Nail (InterTAN) for the Surgical Management of Intertrochanteric Fractures of the Hip in Indians

**DOI:** 10.7759/cureus.45942

**Published:** 2023-09-25

**Authors:** Siva Mahesh S, Nirav R Gupta, Prasad Soraganvi

**Affiliations:** 1 Department of Orthopaedics, St. Peter's Medical College, Hospital and Research Institute, Hosur, IND

**Keywords:** functional outcome, cephalomedullary nails, intertrochanteric fracture, intertan nail, short versus long nails

## Abstract

Introduction: With an increasing life expectancy, there has been an increase in the incidence of intertrochanteric fractures. These fractures in the elderly are disabling and have a tremendous impact on the healthcare system. Despite substantial improvements in implant design and surgical techniques, high failure rates have been observed, varying with the severity of the fracture and the type of fixation. Intramedullary nails have become popular in recent times, especially in unstable fractures. The purpose of the present study is to compare the functional outcomes and complications of long versus short InterTAN cephalomedullary nails (Smith & Nephew, Memphis, Tennessee) used for intertrochanteric fracture fixation.

Materials and methods: All patients who had intertrochanteric fractures classifiable as AO OTA (Arbeitsgemeinschaft für Osteosynthesefragen/Orthopaedic Trauma Association) 31-A and were treated with either a short InterTAN nail (SIN) or long InterTAN nail (LIN) between March 2017 and March 2020 were retrospectively assessed. AO subtype A1 fractures are considered to be stable whereas subtype A3 fractures are considered unstable. The stability of subtype A2 fractures is variable depending on the amount of posteromedial comminution. Both stable and unstable fractures were included. Patients aged above 18 years, who had a normal pre-injury gait and were operated on within seven days of trauma as per the records were included in the study. Postoperatively, functional outcome recorded using the modified Harris hip score (mHHS) was compared. The minimum follow-up period was 24 months.

Results: A total of 89 patients fulfilling the inclusion criteria were included. The mean age was 67.5 ± 8.92 years. Of the patients, 72% were above 60 years of age and 68% of those were females. The mean follow-up period was 31 months (range: 24-54 months). Of the patients, 84.27% sustained fractures after a trivial trauma due to slip and fall at home. All fractures had united at nine to 12 months, except one had a screw cut-out, which required revision surgery. The mean mHHS at three months and nine to 12 months postoperatively was 42.46 ± 3.62 and 87.24 ± 6.44, respectively. The patients who were treated with LIN had a significantly better functional outcome at three-month follow-up (p-value < 0.05); however, post one year, this effect plateaued and no significant difference was seen when comparing SIN with LIN. The results also showed that there was no significant difference in complications among SIN and LIN.

Conclusion: Both LIN and SIN are equally effective for the surgical management of intertrochanteric fractures, and have similar functional outcomes. SIN, however, has shorter surgical procedure time and lesser estimated blood loss. LIN allowed in early recovery evidenced by better Harris hip scores at three months duration, thus improving the quality of life in the initial months post surgery. The choice of implant should be individualized according to fracture anatomy, patients’ needs and expectations, and surgeons’ expertise.

## Introduction

With an increasing life expectancy, there has been an increase in the incidence of intertrochanteric fractures. Epidemiologic studies conducted in Asian countries have shown a high incidence of hip fractures and estimated a further rise in their number in the near future [1].

It is estimated that there is a lifetime risk of 5.6% in men and 20% in women above 50 years of age for hip fractures [2]. These fractures in the elderly are disabling and have a tremendous impact on both the healthcare system and society in general. Despite substantial modifications in implant design and surgical techniques, a 56% failure rate has been observed, varying with the severity of the fracture and the type of fixation [3]. Fixation of intertrochanteric fractures by use of intramedullary nails has become popular in recent times, especially in the unstable type [4].

The InterTAN nail (Smith & Nephew, Memphis, Tennessee) introduced in 2006 was designed specifically to treat intertrochanteric fractures, and claims to have improved biomechanical properties, providing better rotational stability and linear pressurization advantages [5]. Various studies have compared this newer implant with the old standard implants to find out its benefits. Ricci et al., in their retrospective study, found that there was more fracture collapse in patients treated with dynamic hip screw (DHS) or with a single-blade or screw trochanteric entry femoral intramedullary nail (TFN) when compared to those treated with the InterTAN nails [6]. Traditional classified as stable fracture patterns (two-part fracture) were not always stable when treated with TFN or DHS and showed a collapse of more than 10-20 mm [6]. Quartley et al., in their systemic review and meta-analysis, concluded that the use of InterTAN intramedullary nails for unstable intertrochanteric fractures reduces the risk of revision surgery by 64% and implant-related failures by 62% [7]. There were no differences in healing time, non-unions, infections, or Harris hip scores between the various intramedullary nail designs [7].

However, there is a paucity of literature comparing the short InterTAN nail (SIN) with the long InterTAN nail (LIN), and therefore, the purpose of the present study is to compare the functional outcomes, radiological outcomes (implant failure, screw cut-out, etc.), and complications of short versus long InterTAN cephalomedullary nail used for intertrochanteric fracture fixation.

## Materials and methods

This retrospective study was conducted in a tertiary care center in the southern part of India. We reviewed all patients who had intertrochanteric fractures classifiable as AO OTA (Arbeitsgemeinschaft für Osteosynthesefragen/Orthopaedic Trauma Association) 31-A and were treated with either a SIN or LIN between March 2017 and March 2020. AO subtype A1 fractures are considered to be stable whereas subtype A3 fractures are considered unstable. Stability of subtype A2 fractures is variable depending on the amount of posteromedial comminution. Both stable and unstable fractures were included in this study. Injury due to slip and fall while walking or at home was considered a low-velocity injury and that after falling from a height or road traffic accident was considered a high-velocity injury. All patients above 18 years of age (skeletal maturity), who had a normal pre-injury gait and were operated on within seven days of trauma as per the records were included in the study. Patients with open injury, subtrochanteric fractures, pathological fractures, previous surgery around the hip joint, and previous failed surgeries were excluded from the study. Patients who had a follow-up of less than two years were also excluded.

Descriptive surgical procedure and mobilization

All the cases were performed by a single senior orthopedic surgeon as per the standard protocols. There are no clear-cut indications or guidelines for short versus long nail use in intertrochanteric fractures. Some surgeons prefer short nails in very high-risk patients due to the estimated short surgical time. Long nails are preferred in unstable types of fractures. The use of short or long nails was decided by the operating surgeon based on his experience. Patients were positioned supine on a fracture table. The normal leg was flexed and abducted at the hip so as to have easy access for fluoroscopy. The fracture reduction was attained by giving traction in neutral and rotating the limb internally or externally depending on the nature of the fracture and checked by anteroposterior and lateral views on the image intensifier. All fractures were reduced by the closed or minimally invasive methods. The InterTAN nail was then inserted and under fluoroscopic guidance, proximal integrated compression screws were passed to achieve compression at the fracture site. Distal locking was performed using a jig in short nails and a free-hand technique in long nails. Postoperatively, the patients were mobilized in bed on the first postoperative evening. The patients were allowed partial weight-bearing (25% of body weight) ambulation with the help of a walker as soon as pain and the general condition permitted. Full weight bearing was commenced after fracture healing was demonstrated on radiographs.

Regular follow-up data at three months, nine to 12 months, and two years were assessed. Total surgical time, estimated blood loss, duration of hospital stay post procedure, and complications reported intraoperatively, immediately post surgery, or in the follow-up period were reviewed from the case sheets. Follow-up X-rays were studied to assess radiological outcomes. Postoperatively functional outcome recorded using modified Harris hip score (mHHS) was compared.

Statistical analysis

Descriptive statistics with demographic details of study patients were calculated using Microsoft Excel version 2013 (Microsoft Corporation, Redmond, WA) and SPSS (IBM Corp., Armonk, NY). Unpaired t-test and chi-square test were used to compare the duration of surgery, length of hospital stay, estimated blood loss, and functional outcome between the patients treated by a SIN versus a LIN for intertrochanteric fractures. Chi-square analysis was used to ascertain the statistical significance of the differences between categorical variables, and an unpaired t-test was used for continuous variables. A p-value of <0.05 was considered to be significant.

## Results

**Figure 1:**
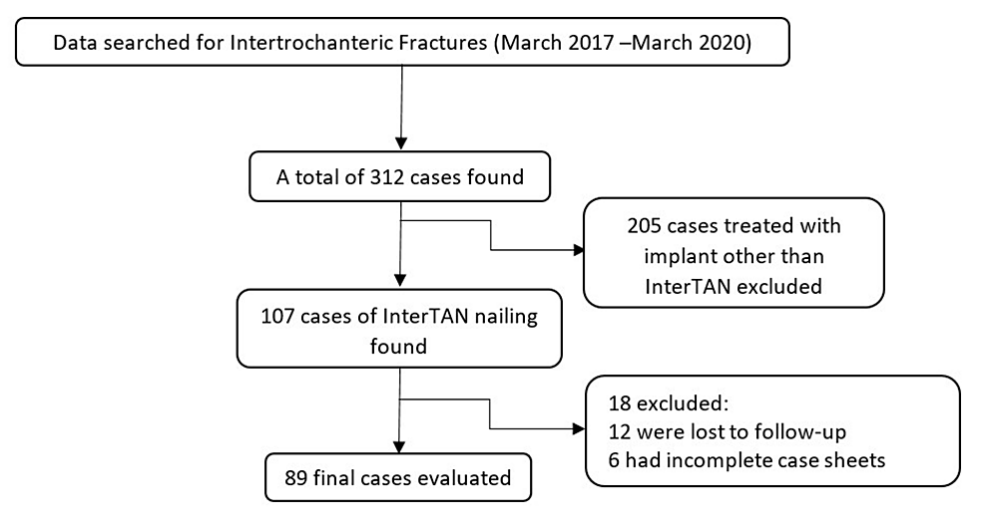
Inclusion and exclusion of cases

**Figure 2:**
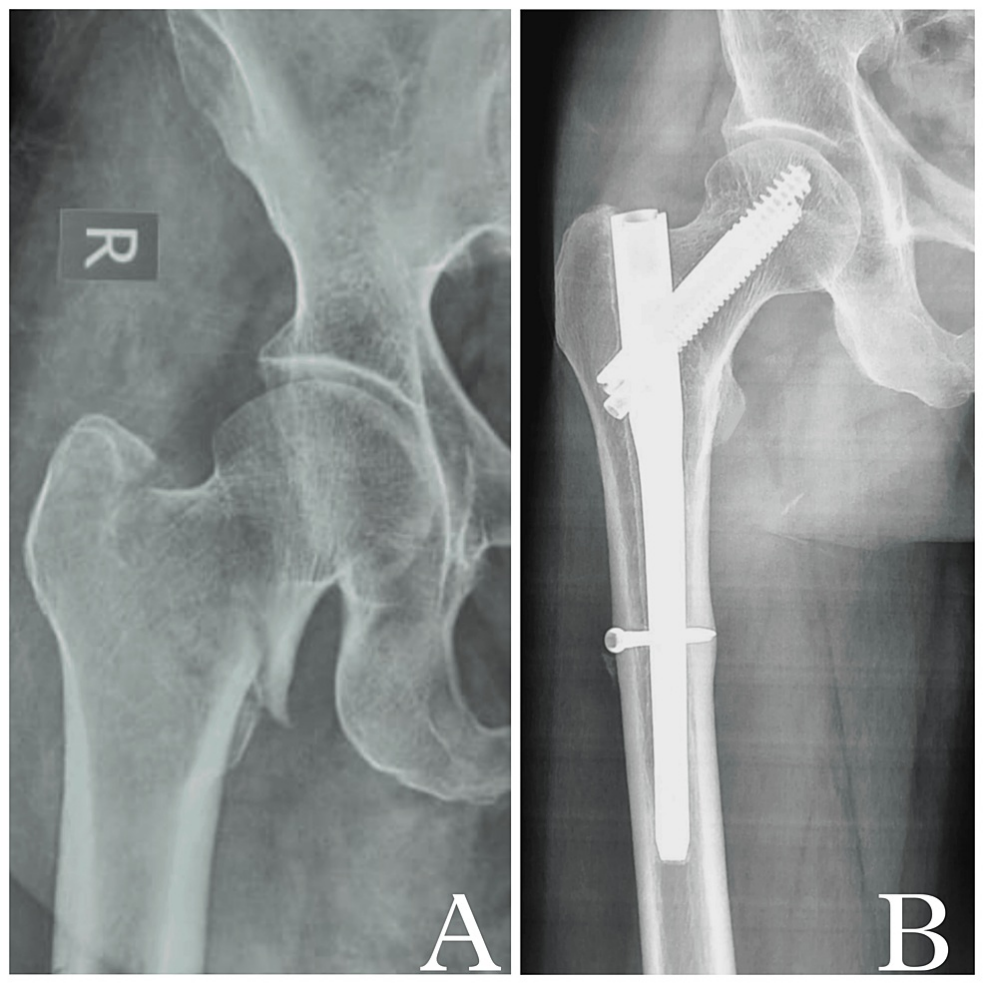
Intertrochanteric fracture fixation using short InterTAN nail (A) Preoperative radiograph showing right intertrochanteric fracture. (B) Two-year follow-up X-ray showing fracture union. Note there is no implant loosening, screw back-out, or collapse of fracture.

**Figure 3:**
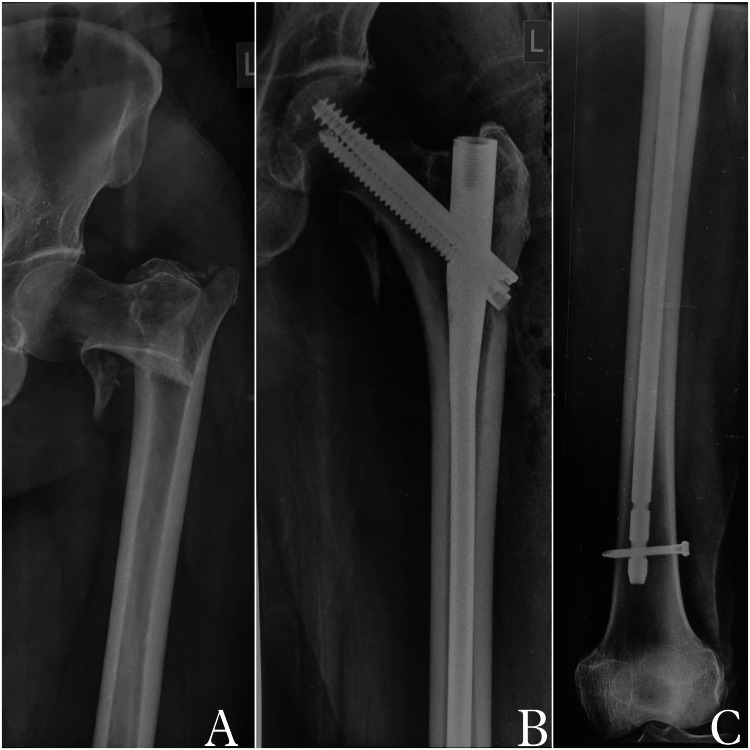
Intertrochanteric fracture fixation using long InterTAN nail (A) Preoperative X-ray showing left intertrochanteric fracture. (B) Two-year follow-up radiograph showing united fracture and long InterTAN nail in situ. (C) Distal locking of InterTan nail seen.

A total of 89 patient case sheets meeting the inclusion criteria were identified (Figure 1) and divided into two groups: Group A comprising 46 patients (51.69%) who were treated with SIN (Figure 2), and Group B comprising 43 patients (48.31%) who were treated with LIN (Figure 3) for AO OTA 31-A trochanteric fractures. Both stable and unstable fractures were included. Details and distribution among the groups with regard to subtypes have been enlisted in Table 1. In our study, the minimum age of the patients was 45 years and the maximum age was 87 years with a mean age of 67.54 ± 8.92 years. The mean follow-up period was 31 months (range: 24-54 months). Demographic data between the two groups are shown in Table 1.

**Table 1:** Distribution of descriptive data across the two groups (short InterTAN vs. long InterTAN) * P-value > 0.05, which was not significant.

Descriptive parameters	Short InterTAN (n = 46)	Long InterTAN (n = 43)	Total (n = 89)	p-value
Age, n (%)				0.362*
<60	13 (28.26)	12 (27.91)	25 (28)	
>60	33 (71.73)	31 (72.09)	64 (72)	
Mean age (years)(mean ± SD)	64.2 ± 11.05	70.88 ± 10.03		0.319*
Gender, n (%)				0.453*
Male	18 (39.13)	11 (25.58)	29 (32.58)	
Female	28 (60.87)	32 (74.42)	60 (67.41)	
Side of injury, n (%)				0.571*
Right	22 (47.83)	20 (46.51)	42 (47.19)	
Left	24 (52.17)	23 (53.49)	47 (52.81)	
Mode of injury, n (%)				0.123*
High velocity	5 (10.87)	9 (20.93)	14 (15.73)	
Low velocity	41 (89.13)	34 (79.07)	75 (84.27)	
Type of fracture (AO Classification), n (%)				0.096*
A1	18 (39.13)	10 (23.25)	28 (31.46)	
A2	21 (45.65)	23 (53.49)	44 (49.44)	
A3	7 (15.22)	10 (23.25)	17 (19.1)	
Stability of fracture, n (%)				0.072*
Stable	27 (58.7)	19 (44.18)	46 (51.69)	
Unstable	19 (41.3)	24 (55.81)	43 (48.31)	

Most of the patients (84.27%) had a low-velocity injury and AO OTA 31-A2 was the most common type of fracture seen (49.44%). The distribution of type of fracture across the two groups was similar (p = 0.096).

The duration of surgery was calculated as the total time taken from skin incision to final wound closure. The mean duration of surgery in our study was found to be 84.1 ± 21.87 minutes. The minimum duration was 48 minutes and the maximum was 123 minutes. SIN had a significantly lower duration of surgery (p-value < 0.01) when compared to long InterTAN nailing (Table 2). The mean estimated blood loss was 124.5 ± 48.5 ml in our study. Distribution across the two groups is shown in Table 2. The total number of days the patient was admitted to the hospital post procedure was noted as the duration of the hospital stay. The mean length was 5.3 ± 1.64 days in this study. However, no significant difference was found in the duration of hospital stay among the two groups (Table 2).

**Table 2:** Comparison of operative data, stay duration, and complications across the two groups (short InterTAN nail vs. long InterTAN nail) * P-value < 0.05 calculated by unpaired t-test. A statistically significant difference was found.

Parameter	Short InterTAN nail (n = 46)	Long InterTAN nail (n = 43)	p-value
Duration of surgery, min (mean ± SD)	70.4 ± 19.19	97.8 ± 14.75	<0.01*
Estimated blood loss, ml (mean ± SD)	110 ± 33.64	140 ± 52.4	<0.01*
Length of stay, days (mean ± SD)	5.28 ± 0.31	5.32 ± 1.77	0.993
Screw cut out, n (%)	1 (2.17)	-	0.312
Infection, n (%)	1 (2.17)	2 (4.65)	0.342

All fractures had united at nine to 12 months, which was evaluated on plain radiographs, except the one with screw cut-out, which required revision surgery. Due to the retrospective nature of the study and the non-availability of X-rays at frequent intervals, it was not possible to calculate the mean time to union and delayed union and compare them among the two groups. A total of three patients had an infection. Two were treated completely by prolonged use of intravenous antibiotics. One, having a deep infection, required surgery for wound debridement and the use of local antibiotic-eluting Stimulan beads for infection control. The implant was retained in this case. One patient having a screw cut-out required revision surgery and a total hip replacement was performed. No deep vein thrombosis, thromboembolism, or peri-implant fractures were reported. There was no implant loosening or breakage. There were a total of five mortalities, two (4.34%) in the SIN group and three (6.97%) in the LIN group. However, no significant difference was found between the two groups in terms of mortality rate (p > 0.05).

The mean mHHS at three months and at nine to 12 months postoperatively was 42.46 ± 3.62 and 87.24 ± 6.44, respectively. The patients who were treated with long cephalomedullary InterTAN nails had a significantly better functional outcome at three months follow-up (p-value < 0.05); however, after one year, this effect plateaued and no significant difference was seen when comparing SIN with LIN (Table 3). The p-value was 0.478, which was not significant, and it suggested that both groups had comparable functional results at the final follow-up.

**Table 3:** Comparison of modified Harris hip score (mHHS) at follow-up between short InterTAN nailing and long InterTAN nailing * Statistically significant difference calculated by unpaired t-test.

Modified Harris hip score	Short InterTAN nail (n = 46)	Long InterTAN nail (n = 43)	p-value
At 3 months (mean ± SD)	40.96 ± 3.08	44.19 ± 3.55	<0.05*
At 9-12 months (mean ± SD)	85.76 ± 6.58	88.32 ± 6.17	0.162
At 2 years (mean ± SD)	87.54 ± 5.89	89.36 ± 6.87	0.478

The patient-reported functional outcome calculated by the mHHS showed that 12 patients (13.48%) had excellent scores and 59 patients (66.29%) had good scores. However, 10 patients (11.23%) and eight patients (8.99%) had fair and poor mHHS, respectively, and did not show satisfactory results. The distribution across the two groups is shown in Table 4, which was not statistically different.

**Table 4:** Functional outcome at the final follow-up between the two groups (short InterTAN nail vs. long InterTAN nail) * P-value > 0.05. The difference was not significant. mHHS: modified Harris hip score.

Outcome (mHHS)	Short InterTAN nail (n = 46)	Long InterTAN nail (n = 43)	Total (n = 89)	p-value
Excellent, n (%)	5 (10.87)	7 (16.28)	12 (13.48)	0.816*
Good, n (%)	30 (65.22)	29 (67.44)	59 (66.29)	0.738*
Fair, n (%)	6 (13.04)	4 (9.3)	10 (11.23)	0.189*
Poor, n (%)	5 (10.87)	3 (6.98)	8 (8.99)	0.594*

## Discussion

The functional aspect plays a very important role in the evaluation of any surgical outcome and, unfortunately, it is not reported in many of the studies that have compared short cephalomedullary nails with long cephalomedullary nails. The primary objective of this study was thus to report the functional outcome with either length of the InterTAN cephalomedullary implant. In this study, LIN allowed in early recovery evidenced by better Harris hip scores at three months duration, thus improving the quality of life in the initial months post surgery. However, after one year, the mHHS was similar in both groups and it stayed statistically insignificant till the final follow-up (minimum of two years). Of the patients treated with InterTAN nail, 79.77% had excellent to good results with similar distribution across the two groups in this current study.

The majority of the patients belonged to the geriatric age group with the mean age being 67.5 ± 8.92 years. Of the patients, 72% were above 60 years of age and 68% of these were females. Of the patients, 84.27% sustained this injury after a trivial trauma. The results showed no significant difference in complications among the SIN and LIN groups. However, three patients had an infection and one patient had a nonunion secondary to a screw cut-out (AO OTA 31-A3 type) requiring revision.

A large series that studied 559 patients compared long and short Gamma nails used for intertrochanteric fracture fixation and found a screw cut-out rate of 2.86%. However, there was no difference found between the two groups [8]. Two other retrospective studies evaluating Gamma 3 and TFN found no difference in screw cut-outs among the long and the short nail groups [9,10]. Our study based on the use of SIN and LIN also found no statistical difference with regard to screw cut-outs. There was no implant-related complication seen.

The short intramedullary nails had the advantage of having a jig for distal locking hence reducing the surgical time and radiation exposure [11]. However, the risk of peri-implant fractures around the nail tip is high, especially in patients having osteoporosis [12]. It is reported to be around 0% to 8% in the literature [13]. Theoretically, any long nail fills the entire medullary canal reducing the chances of these pathological fractures [14].

Norris et al. showed a fracture rate of 1.7% in short nails as compared to 1.1% in long nails; the difference of which was not statistically significant [15]. Other studies also showed a similar result when comparing the occurrence of fractures around the nail [8-10]. Our study did not have any peri-implant fracture and there is no conclusive evidence in the literature regarding the same.

Previous studies [8-10] have shown consistently longer surgical duration for long nails, as was found in the current study. As discussed, this can be attributed to the fact that short nails have a jig for distal locking whereas in long nails, distal locking has to be done by free hand perfect circle technique. There is an improvement in operating room efficacy and patient safety with shorter operative time. Elderly patients who usually have multiple comorbidities have reduced postoperative complications and smoother rehabilitation if associated with shorter operative time [16,17]. A smaller traction time also reduces the associated risk of pressure injury to the pudendal nerve caused by the perineal post [18]. Kleweno et al. [8] found that when no distal locking was done for long nails, the operative time was similar to that of short nails.

Radiation exposure due to intraoperative imaging is a very important consideration for the safety of the operating room staff. Okcu et al. [19], in a prospective study, reported an intraoperative imaging time of 58.6 seconds for short nails as compared to 75.3 seconds for long nails. This finding was again found to be associated with freehand distal locking in long nails. The use of jigs in shorter nails made it possible to put the distal screw without any use of intraoperative imaging. Our study could not evaluate this due to a lack of available data.

As per a study by Frisch et al. [20], the estimated blood loss (208.1 ± 116.9) for LIN was significantly higher than SIN (161.4 ± 122.4). A similar consensus was found in the literature and reported in a systemic review and meta-analysis by Tan et al. [21]. They reported an average excessive loss of 28.81 ml in the long nail group when using the Gamma nail or TFN and this could be attributed to reaming of the femoral canal done for insertion of a long intramedullary nail. Our study also showed that SIN was associated with significantly lesser estimated blood loss. Lesser blood loss in an elderly patient is beneficial for quicker recovery and healing.

Fewer studies have recorded the duration of hospital stay when comparing the short and the long intramedullary nails for intertrochanteric fractures. Dunn et al. [22] reported a mean duration of seven days versus 7.3 days for short and long nails, respectively, whereas Guo et al. [23] reported a hospital stay duration of 12.7 days versus 12.9 days. Both of these studies did not show any significant difference in stay duration, as was seen in our study (5.28 vs. 5.32 days). Duration of hospital stay was not found to be associated with the length of the nail used.

A study by Frisch et al. [20] has compared SIN with LIN. However, this study has a short follow-up period of a minimum of eight weeks and did not report functional outcomes. Our study has compared the functional outcome among the SIN and LIN groups using mHHS. The mHHS was 87.54 ± 5.89 in the SIN group and 89.36 ± 6.87 in the LIN group at the final follow-up, the difference of which was found to be statistically insignificant (p = 0.478).

Limitations of the study

There is a potential selection bias due to the retrospective nature of the study. There was no randomization protocol and the patients were chosen for a particular group as per the surgeon’s preference. These study findings can be confirmed and explored further by increasing the sample size, and data can be pooled from other centers, thus making it a multi-centric study. Due to the non-availability of X-rays at frequent intervals, it was not possible to calculate the mean time to union and delayed union and compare them.

## Conclusions

Both LIN and SIN are equally effective for the surgical management of intertrochanteric fractures and have similar functional outcomes in the long term. SIN, however, has shorter surgical procedure time and lesser estimated blood loss. LIN allowed in early recovery evidenced by better Harris hip scores at three months duration, thus improving the quality of life in the initial months post surgery. The choice of implant should be individualized according to fracture anatomy, patients’ needs and expectations, and surgeons’ expertise.

## References

[1] Kim JW, Kim TY, Ha YC, Lee YK, Koo KH (2015). Outcome of intertrochanteric fractures treated by intramedullary nail with two integrated lag screws: a study in Asian population. Indian J Orthop.

[2] Dhanwal DK, Dennison EM, Harvey NC, Cooper C (2011). Epidemiology of hip fracture: worldwide geographic variation. Indian J Orthop.

[3] Haidukewych GJ, Israel TA, Berry DJ (2001). Reverse obliquity fractures of the intertrochanteric region of the femur. J Bone Joint Surg Am.

[4] Roberts KC, Brox WT, Jevsevar DS, Sevarino K (2015). Management of hip fractures in the elderly. J Am Acad Orthop Surg.

[5] Ruecker AH, Rupprecht M, Gruber M, Gebauer M, Barvencik F, Briem D, Rueger JM (2009). The treatment of intertrochanteric fractures: results using an intramedullary nail with integrated cephalocervical screws and linear compression. J Orthop Trauma.

[6] Ricci MJ, McAndrew CM, Miller AN, Kamath G, Ricci WM (2019). Are two-part intertrochanteric femur fractures stable and does stability depend on fixation method?. J Orthop Trauma.

[7] Quartley M, Chloros G, Papakostidis K, Saunders C, Giannoudis PV (2022). Stabilisation of AO OTA 31-A unstable proximal femoral fractures: does the choice of intramedullary nail affect the incidence of post-operative complications? A systematic literature review and meta-analysis. Injury.

[8] Kleweno C, Morgan J, Redshaw J (2014). Short versus long cephalomedullary nails for the treatment of intertrochanteric hip fractures in patients older than 65 years. J Orthop Trauma.

[9] Boone C, Carlberg KN, Koueiter DM (2014). Short versus long intramedullary nails for treatment of intertrochanteric femur fractures (OTA 31-A1 and A2). J Orthop Trauma.

[10] Hou Z, Bowen TR, Irgit KS, Matzko ME, Andreychik CM, Horwitz DS, Smith WR (2013). Treatment of pertrochanteric fractures (OTA 31-A1 and A2): long versus short cephalomedullary nailing. J Orthop Trauma.

[11] Kreder HJ (2013). Principles and evidence: the optimal treatment of pertrochanteric hip fractures: commentary on an article by Kjell Matre, MD, et al.: "TRIGEN INTERTAN intramedullary nail versus sliding hip screw. A prospective, randomized multicenter study on pain, function, and complications in 684 patients with an intertrochanteric or subtrochanteric fracture and one year of follow-up". J Bone Joint Surg Am.

[12] Rosenblum SF, Zuckerman JD, Kummer FJ, Tam BS (1992). A biomechanical evaluation of the Gamma nail. J Bone Joint Surg Br.

[13] Erez O, Dougherty PJ (2012). Early complications associated with cephalomedullary nail for intertrochanteric hip fractures. J Trauma Acute Care Surg.

[14] Kanakaris NK, Tosounidis TH, Giannoudis PV (2015). Nailing intertrochanteric hip fractures: short versus long; locked versus nonlocked. J Orthop Trauma.

[15] Norris R, Bhattacharjee D, Parker MJ (2012). Occurrence of secondary fracture around intramedullary nails used for trochanteric hip fractures: a systematic review of 13,568 patients. Injury.

[16] Belmont PJ Jr, Goodman GP, Waterman BR, Bader JO, Schoenfeld AJ (2014). Thirty-day postoperative complications and mortality following total knee arthroplasty: incidence and risk factors among a national sample of 15,321 patients. J Bone Joint Surg Am.

[17] Schoenfeld AJ, Carey PA, Cleveland AW 3rd, Bader JO, Bono CM (2013). Patient factors, comorbidities, and surgical characteristics that increase mortality and complication risk after spinal arthrodesis: a prognostic study based on 5,887 patients. Spine J.

[18] Brumback RJ, Ellison TS, Molligan H, Molligan DJ, Mahaffey S, Schmidhauser C (1992). Pudendal nerve palsy complicating intramedullary nailing of the femur. J Bone Joint Surg Am.

[19] Okcu G, Ozkayin N, Okta C, Topcu I, Aktuglu K (2013). Which implant is better for treating reverse obliquity fractures of the proximal femur: a standard or long nail?. Clin Orthop Relat Res.

[20] Frisch NB, Nahm NJ, Khalil JG, Les CM, Guthrie ST, Charters MA (2017). Short versus long cephalomedullary nails for pertrochanteric hip fracture. Orthopedics.

[21] Tan GK, Chong CS, Bin Abd Razak HR (2021). Clinical outcomes following long versus short cephalomedullary devices for fixation of extracapsular hip fractures: a systematic review and meta-analysis. Sci Rep.

[22] Dunn J, Kusnezov N, Bader J, Waterman BR, Orr J, Belmont PJ (2016). Long versus short cephalomedullary nail for trochanteric femur fractures (OTA 31-A1, A2 and A3): a systematic review. J Orthop Traumatol.

[23] Guo XF, Zhang KM, Fu HB, Cao W, Dong Q (2015). A comparative study of the therapeutic effect between long and short intramedullary nails in the treatment of intertrochanteric femur fractures in the elderly. Chin J Traumatol.

